# Cutaneous Tuberculosis: Clinicopathologic Arrays and Diagnostic Challenges

**DOI:** 10.1155/2018/7201973

**Published:** 2018-07-09

**Authors:** Priyatam Khadka, Soniya Koirala, Januka Thapaliya

**Affiliations:** ^1^Department of Microbiology, Tribhuvan University Teaching Hospital, Kathmandu, Nepal; ^2^M.Sc. Medical Microbiology, Tri-Chandra Multiple Campus, Tribhuvan University, Nepal; ^3^Department of Dermatology and Venerology, Tribhuvan University Teaching Hospital, Kathmandu, Nepal; ^4^M.D. Dermatology, Maharajgunj Medical Campus, Tribhuvan University, Nepal

## Abstract

The clinicopathological manifestations of cutaneous tuberculosis are diverse. The precise diagnosis is often overlooked, due to clinical presentations as those of cutaneous diseases with different etiology and the relative paucity of the pathogens in the lesions. Meanwhile, almost all of the diagnostic methods confer lower sensitivity and specificities which augments further diagnostic challenges. This article revises the current scenario of the disease's physiopathology and underscores clinicopathological challenges, due to multifaceted presentations of cutaneous tuberculosis, in the diagnosis.

## 1. Background

Cutaneous tuberculosis is a relatively uncommon, comprising 1-1.5% of all extrapulmonary tuberculosis manifestations, which manifests only in 8.4-13.7% of all tuberculosis cases [1]. Although rare, given its global prevalence, it is imperative for the clinicians to distinguish the many clinical variants of cutaneous tuberculosis and the masquerading infections—granulomatous syphilis, discoid lupus erythematosus, psoriasis, tuberculoid leprosy, sarcoidosis, actinomycosis, mycetoma, bacterial abscesses, and other skin infections—to preclude missed or delayed diagnosis [2, 3]. Most of the diagnostic methods for cutaneous tuberculosis confer lower sensitivity and specificities. Therefore, the physicians must resort to every possible test along with broad clinical consideration; hence the summation of positive rudiments would be auxiliary in precise diagnosis.

## 2. Epidemiology

Tuberculosis represents a major public health problem in Southeast Asia, since a larger proportion (45%) of total estimated 10.4 million infective cases were listed in the region [4]. Compiling the toll death rate, Southeast Region and African Regions accounted for 85% of total death due to tuberculosis [4]. TB ranks the 6th leading cause of death in Nepal [5]. The prevalence study was not done in Nepal due to impassiveness of government participation in the health sector; however, annually, 34,122 cases of tuberculosis were reported to NTP [6].

Tuberculosis is endemic in Nepal; limited cases of cutaneous tuberculosis were reported, however. The incidence of cutaneous tuberculosis in Central Nepal was reported as 0.1%; nonetheless, the exact incidence is still anonymous over the country. The clinicoepidemiological study done in Nepal by Dwari et al. 2010 revealed tuberculosis verrucous (48%) as predominant clinical type [7]; however, on referencing to earlier studies, Lupus vulgaris was the most common (64%), followed by tuberculosis verrucosa cutis (19%) and papulonecrotic tuberculid (4%) [8]. Ironically, cases of cutaneous multidrug resistant tuberculosis (MDR-TB)—resistant with at least two of the most potent first-line anti-TB medications, isoniazid and rifampicin—and XDR-TB—MDR strains that are resistant to fluoroquinolones plus one of the injectables such as kanamycin, amikacin, and capreomycin—have also been reported from India and China abutting Nepal [9–11]. Nevertheless, the exact epidemiological entity of perchance MDR/XDR cutaneous tuberculosis cases is still unbeknownst or unreported from Nepal.

## 3. Etiological Agent

The main etiological agent of the Cutaneous tuberculosis is* Mycobacterium tuberculosis*—occasionally* M. bovis* or BCG vaccine (an attenuated strain of* M. bovis*) [12, 13].

*Mycobacterium tuberculosis* is a straight or slightly bent (rod-shaped), nonmotile, nonsporulated, bacillus, being 1 to 10 *μ*m long and 0.2 to 0.6 *μ*m wide; its most important feature is acid-fastness due to high lipid content in the cell wall. Approximately there are 4000 genes with most of them involved in the mechanism of immune system invasion and 200 of them for lipid metabolism; consequently, the pathogen is able to survive both inside and outside the phagocytic cells [14]. Meanwhile, as lipids are the main energy source of* Mycobacterium tuberculosis*, the pathogen is directly responsible for multiplying in host tissue and forming cellular walls [14, 15].

## 4. Route of Infection

Cutaneous tuberculosis can be acquired from hematogenous or lymphatic dissemination of a pulmonary focus or by direct inoculation. The pivotal factor for the clinical presentations prior to contact with bacilli is the host natural immune response, however.

Exogenous infection occurs with direct inoculation of bacilli into the skin of predisposed individuals (tuberculous chancre, tuberculosis verrucosa cutis) [1].

Endogenous infection is secondary to a preexisting primary focus and may result from contiguous (orificial tuberculosis, scrofuloderma), hematogenous (acute miliary tuberculosis, tuberculous gumma, and lupus vulgaris), or lymphatic dissemination (lupus vulgaris) [2, 16].

## 5. Classifications of Cutaneous Tuberculosis Based on a Load of Pathogens

Based on a load of the pathogens on skin, the tuberculosis variant can be classified into two broad categories.

Multibacillary forms (easily detected in cutaneous tissue) include tuberculous chancre, scrofuloderma, orificial tuberculosis, acute miliary tuberculosis, and tuberculous gumma [17, 18].

Paucibacillary forms (bacilli being sparse) include TB verrucosa cutis, tuberculoid, and lupus vulgaris [17, 18].

## 6. Clinical Manifestations of Cutaneous Tuberculosis

Cutaneous tuberculosis exhibits diverse clinical manifestations: inflammatory papules, verrucous plaques, suppurative nodules, chronic ulcers, and other atypical lesions [19].

## 7. Exogenous Cutaneous Tuberculosis

### 7.1. Tuberculosis Chancre

The direct inoculations of Mtb in the skin from the traumatic injuries or surgical procedures performed with unsterilized materials and even after tattoos or body piercing lead to acquired tuberculosis chancre. Progressing from a firm, painless, reddish-brown, slow-growing papule, or nodule, after 2 to 4 weeks it develops into the friable ulcers—tendency to bleed with a granular surface [20]. Furthermore, the bacilli disseminate to regional lymph nodes via lymph.

Presumptive identification can be done with histopathological examinations, where the acute neutrophilic inflammatory reaction prolific in AFB and necrotic areas are usually noticed [16]. Sequentially, the lesion acquires a granulomatous form with enlarged giant cells after 3 to 6 weeks with the reduced number of bacilli [20].

### 7.2. Tuberculosis Verrucosa Cutis

Tuberculosis verrucosa cutis, the usual exogenous form of tuberculosis, is more common in an anatomist, physicians, and bare-footed children of tropical zones, since the infection proceeds with an injured dermal layer [1]. The lesions—solitary, painless, and without adenopathy—are more seen commonly in the extremities prone to traumas [16]. The lesions jerk as erythematous papules to verrucous plaques with peripheral extension.

## 8. Endogenous Tuberculosis

### 8.1. Scrofuloderma

Scrofuloderma, also called colliquative cutis, is a common form of cutaneous tuberculosis; it results from direct extension from an underlying tuberculosis lesion in lymph node, bone, joints, or testicles [1, 2]. The neck, axillae, and groin are often involved, with the cervical lymph nodes as a common source of infection [1]. Early lesions appear as firm, painless, subcutaneous, and red-brown nodules which advanced to ulcers and discharging sinus [21]. Spontaneous healing may occur, leaving keloid scars, retractions, and the atrophic sequel [21].

### 8.2. Orificial Tuberculosis

Orificial tuberculosis—a very rare form of cutaneous tuberculosis— is clinically characterized by ulcerations at mucocutaneous orifices including mouth, nose, perianal region, and genitalia and adjacent skin, usually advanced form of lungs, intestinal, or genitourinary tuberculosis [22]. The lesions, about 1 to 3 cm in diameter, appear as friable, painful erythematous-to-yellowish papules and nodules, which may advance to painful ulcers [16]. Edema and inflammation are obvious in perilesional tissue.

### 8.3. Lupus Vulgaris

Lupus vulgaris is the most common form of cutaneous tuberculosis in Europe, India, and Nepal [8, 13, 16]. It is a chronic, progressive, paucibacillary form of cutaneous tuberculosis which occurs primarily in the previously sensitized individual [23, 24]. The infection occurs endogenously via lymphohematogenous route and occasionally via exogenous route—with drainage scar of scrofuloderma [25].

**Figure 1:**
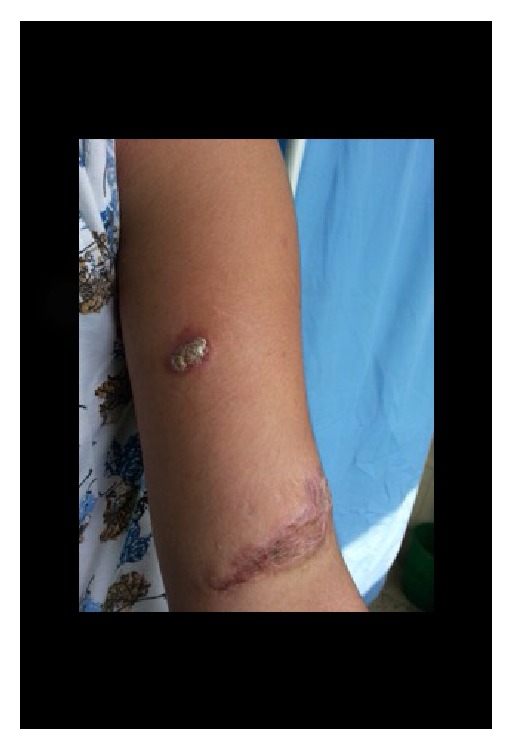
Erythematous plaque (2*∗*1 cm) of lupus vulgaris on right forearm of a 17-year-old female with a history of trauma forming a linear scar (4*∗*2 cm), visiting TUTH.

**Figure 2:**
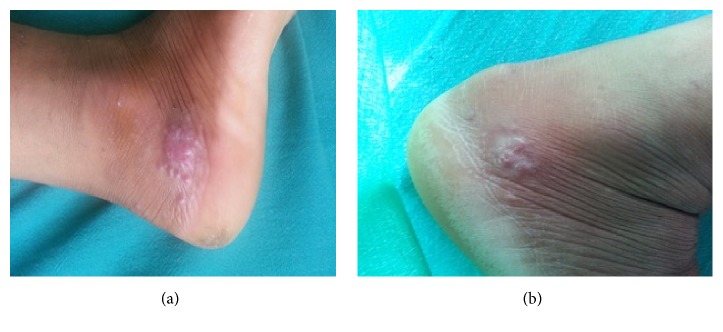
(a) Multiple erythematous papules of lupus vulgaris below lateral malleolus of right foot of 34-year-old female with a history of trauma on the right foot working in field 6 months earlier (before treatment). (b) The erythematous plaque reduced but did not resolve completely after antitubercular therapy; antitubercular therapy continued for three more months.

The most typical clinical feature of lupus vulgaris is a papulotubercular lesions commonly on the legs and buttocks, which eventually coalesce into a plaque (Figures 1, 2(a), and 2(b)) [12]. The plaques grow peripherally, with serpiginous or verrucous borders, accompanied by central discoloration and atrophy [25]. Besides, the classic appearance is described as “apple jelly nodules” observed on diascopy [24, 26].

### 8.4. Tuberculous Gumma

Tuberculous gumma, also known as metastatic tuberculosis abscess, is an outcome of hematogenous dissemination of mycobacteria from primary focus especially in an immunocompromised host, scarcely in an immunocompetent host too [17, 27]. Clinically it may bear a semblance to scrofuloderma; few lesions affecting trunks and extremities with inconsistent subcutaneous nodules having tendency to ulcerate and drain caseous secretion are seen in tuberculous gumma [23].

### 8.5. Acute Miliary Tuberculosis

It is a rare presentation of cutaneous tuberculosis predominantly in severely immunocompromised host, demonstrating anergy. The bulk of cases have been increasing primarily due to coinfection with HIV with declining CD4 count below 100 cells/*µ*L [28]. Clinically, diverse cutaneous lesions—erythema and erythematous-whitish or erythematous-purplish papules—may be noticed which later on break to form umbilication and crust formation leaving hypochromic scars [17].

## 9. Tuberculids

Tuberculids are acute or chronic cutaneous forms of tuberculosis, appearing with diverse clinical forms, having a propensity of hyperergic expressions, active TB, or disseminated forms [20]. The discrete relationship between tuberculids and TB continues to be debated because the clinical forms usually have a symmetrical distribution, tuberculous involvement (usually inactive) of viscera or lymph nodes, and the absence of AFB (low positivity to culture and PCR) in the lesions [16, 26].

### 9.1. Papulonecrotic Tuberculids

Papulonecrotic tuberculids are the commonly observed form of cutaneous in children and young people [29]. They appear as painless, symmetrical erythematous, or violaceous papulonodular lesions noted particularly around the face, ears, extensor areas of the trunk, extremities, and buttocks, leaving a depressed scar [26].

### 9.2. Lichen Scrofulosorum

Lichen scrofulosorum is an eruption of multiple, small, grouped, asymptomatic, firm, perifollicular, lichenoid papules or plaques often affecting children and adults with underlying diseases of bone and lymph nodes [16, 26]. The dermatosis leaves no scar after months or years. The onset of this tuberculid was speculated, after BCG vaccinations and in the patient infected with* M. avium-intracellulare* [30].

### 9.3. Erythema Induratum of Bazin

Erythema induratum of Bazin is a granulomatous lobular panniculitis, which appears as erythematous-purplish subcutaneous nodules usually in legs and thighs [26]. The nodules advance few centimeters in diameter forming deep ulcers with caseous discharges and leave pigmented scar without or after successful treatment. The relapse, however, may occur in flares every 3-4 months with similar clinical presentations [1]. Besides, the tendency of coinfectivity with systemic diseases like sarcoidosis is the differential clinical diagnosis of erythema nodosum [16, 21].

## 10. Diagnosis of Cutaneous Tuberculosis

### 10.1. Differential Diagnosis

The precise diagnosis is often significantly deferred and delayed, as cutaneous TB is not routinely considered in the differential diagnosis due to the relative paucity of pathogens in lesions and varied clinical manifestations (Table 1) [2, 16, 19, 31–33]. Hence, differential diagnosis is obligatory for the successful clinical management and treatment.

### 10.2. Laboratory Diagnosis

#### 10.2.1. Tuberculin Skin Test

This technique involves an injection of 0.1 ml tuberculin, purified protein derivatives (PPD) derived from the attenuated strain of* M. tuberculosis*, intradermally, and read after 48 to 72 hours; on positive interpretation, the induration diameter exceeds the measuring of 10mm. The reaction is the classic example of delayed hypersensitivity reaction, where sensitized T-cells by prior infection are recruited thereby releasing the lymphokine [34]. These lymphokines induce indurations through local vasodilation, edema, fibrin deposition, and recruitment of other inflammatory cells to the area [34, 35]. TST has the sensitivity between 33% and 96% and specificity of 62.5% with cutoff 10mm for cutaneous tuberculosis; the sensitivity, however, exceeds 97% in an unvaccinated population [36, 37].

Furthermore, on analyzing clinical forms of cutaneous tuberculosis separately, positivity, intensity of the tuberculin skin test also diverges (Table 2). Conclusively, neither a positive TST necessarily indicates active infection nor a negative TST rules out the infection persistence.

#### 10.2.2. Immunological Tests (Interferon Gamma-Release-Assay)

The FDA approved immunological tests, QuantiFERON and EliSpot, assess sensitizations to* M. tuberculosis* by measuring the amount of INF gamma released by lymphocytes confronted with* M. tuberculosis* specific antigens [16]. The sensitivity and specificity of QuantiFERON are 89% and 99%, respectively, while EliSpot has the sensitivity of 98.8% and a specificity of 100% [38]. Unlike tuberculin skin test (TST), it detects disease in patients who have been vaccinated against BCG (latent infection)—and active infection too.

These tests are still not in routine-practice in our midst, because of high cost and laborious cell extract procedure from culture to antigen preparation (particularly in EliSpot).

#### 10.2.3. Histopathology

Histopathology of a skin biopsy shows granulomatous presentations as those of cutaneous diseases with different etiology—cutaneous leishmaniasis, tuberculid leprosy, superficial granulomatous pyoderma, cutaneous sarcoidosis, lupus miliaris disseminatus faciei, and chromomycosis [16, 19, 33]. Meanwhile, the exact elucidation in diagnosis of cutaneous tuberculosis could not be done; however, the characteristic feature (well-formed granulomas with absence of caseous necrosis, granulomas with caseous necrosis, and the presence of poorly formed granulomas with intense caseous necrosis) would be auxiliary to differentiate types of cutaneous tuberculosis (Table 3) [16, 19, 21, 26, 28, 33, 39].

The equivocal manifestation of cutaneous tuberculosis to correlate the histologic with clinical observations in an evidence-based diagnosis is imperfect and lacking pragmatics.

#### 10.2.4. Diagnosis by Test: Staining and Culture

The mycobacterial cell wall is rich in complex lipids which resists the acid and alcohol; hence the pathogen is termed as acid-fast bacilli (AFB). Staining techniques include Ziehl-Neelsen (common in practice), Kinyoun, and fluorochrome-based techniques with auramine-rhodamine. Microscopic observation of AFB in staining of tissue or secretions enables the empiric therapy if there are sufficient clinical suspicions. However, this does not necessarily suggest the cutaneous tuberculosis, since the other pathogens like* Nocardia*,* Corynebacterium*, nontuberculous mycobacteria, and even artifacts may reveal acid-fast characteristics [38, 40].

Furthermore, the lower sensitivities of staining results in extrapulmonary compared to pulmonary tuberculosis limit the applicability of the test [16, 37, 38]. The cultures of the pathogen,* Mycobacterium tuberculosis*, on specific solid media or by automatic detection of its metabolites in liquid media remain the gold standard method, for identifications and their drug sensitivities. However, the long generation time of the pathogens to grow and lower sensitivity of culture results for lesions and tissue samples attribute further challenges in prompt and accurate diagnosis of cutaneous tuberculosis [16, 38].

#### 10.2.5. Amplifications of Nucleic Acids (PCR)

The detection of Mycobacterium genus using bacterial 16S ribosomal DNA with PCR assays is now termed as a milestone in a diagnosis of pulmonary tuberculosis and several forms of cutaneous tuberculosis. DNA present in a sample of fresh tissues, blood, or a paraffin block even formalin fixed paraffin embedded sections, is amplified and it can then be identified, confirming the presence of mycobacteria [16, 33, 41].

PCR assay has augmented sensitivity and specificity in the diagnosis of cutaneous tuberculosis (Table 4) [42–55]; nevertheless, like other diagnostic approaches it is inconclusive in paucibacillary forms due to unevenly microbial distributions [25, 45].

#### 10.2.6. Genotyping

Genotyping, the recent advance in the diagnosis of cutaneous tuberculosis, has a tendency to separate atypical mycobacteria from Mtb—and detect mutant if it persists inducing drug resistance in the pathogen. The major molecular typing methods—Spoligotyping, MIRU-VNTR (Mycobacterial Interspersed Repetitive Unit-Variable Number Tandem Repeats), and RFLP—detect* Mycobacterium tuberculosis*, DNA, or RNA in clinical specimens by in vitro nucleic acid amplifications, empowering investigations into epidemiology, transmission, and PTB outbreaks [56]. The clinical applicability testing of these genotyping techniques was also accessed in the patients with cutaneous tuberculosis in China by Ziang et al., 2017, with augmented sensitivity and specificity [57].

#### 10.2.7. RFLP (Restriction Fragment Length Polymorphism)

The gold standard in genotyping, IS6110-based restriction fragment length polymorphism (RFLP), has been for more than an epoch; however, it is laborious and costly and requires a large amount of chromosomal DNA [56].

#### 10.2.8. Spoligotyping

Spoligotyping—commonly used to differentiate* Mycobacterium tuberculosis* complex strain—is based on polymorphisms of the chromosomal direct repeat (DR) locus, which contains a variable number of short DRs interspersed with nonrepetitive spacers [56, 57].

#### 10.2.9. Mycobacterial Interspersed Repetitive Unit-Variable Number Tandem Repeat (MIRU-VNTR)

Lately, the International consortium has proposed MIRU-VNTR as a standardized genotyping scheme, with 15- and 24-locus sets proven to have ample discriminatory power for tracing transmission and investigating the phylogenetics of tuberculosis [57].

## 11. Conclusions

In a limelight, almost all of the investigative methods confer lesser sensitivity and specificities for cutaneous tuberculosis, considering atypical erythema nodosum, nonspecific appearance, insufficiently elucidative radio-imaging approaches, histopathology features, and even microbial culture techniques too. The genotyping techniques, nevertheless, could be an assistant to cope with this diagnostic challenge, paradoxically beyond reach to the third world like ours, due to expensive running cost and wanting equipped laboratory setup. In this perspective, the clinicians must resort to every possible test, so that supporting positive rudiments would be ancillary in the early and precise diagnosis of cutaneous tuberculosis.

## Figures and Tables

**Table 1:** Clinical manifestations of cutaneous tuberculosis and its differential diagnosis.

S. N	Classification of cutaneous tuberculosis		Diagnostic considerations
1	Exogenous cutaneous Tuberculosis	Tuberculosis chancre	sporotrichosis, leishmaniasis, atypical mycobacteriosis, syphilis, cat scratch disease and tularemia
		Tuberculosis verrucosa cutis	paracoccidioidomycosis, leishmaniasis, sporotrichosis, tuberculosis verrucosa and chromomycosis. Lobomycosis, atypical mycobacteriosis, hypertrophic lichen planus, verrucous carcinoma, iododerma, bromoderma, verruca vulgaris, keratoacanthoma centrifugum and pyoderma vegetans

2	Endogenous cutaneous tuberculosis	Scrofuloderma	tertiary syphilis, paracoccidioidomycosis, actinomycoses, lymphogranuloma venereum, bacterial abscesses, tumor metastasis, histiocytosis and hidradenitis
		Orificial tuberculosis	bullous diseases, trauma, fungal diseases, syphilis, sarcoidosis, or squamous cell carcinoma
		Lupus vulgaris	basal cell carcinoma, sarcoidosis, discoid lupus erythematosus, Leprosy, Deep Fungal infections
		Tuberculous gumma	leishmania, sporotrichosis, nocardiosis, atypical mycobacteria (*Mycobacterium marinum*), pyogenic infections (*Staphylococcus aureus, Streptococcus*), and deep fungal infections
		Acute miliary tuberculosis	metastatic carcinomas

3	Tuberculids	Papulonecrotic tuberculid	*pityriasis lichenoides et varioliformis acuta* (PLEVA), leukocytoclastic necrotizing vasculitis, pruritus and secondary syphilis
		Lichen scrofulosorum	lichen planus and lichen nitidus, syphilid lichenoides, eczematid, keratosis pilaris, pityriasis rubra pilaris (PRP) and micropapular sarcoidosis
		Erythema induratum of Bazin	erythema nodosum, cutaneous polyarteritis, pancreatic panniculitis, lupus profundus, subcutaneous sarcoidosis and cutaneous T-cell lymphoma

**Table 2:** TST result in different forms of cutaneous tuberculosis.

Clinical forms of cutaneous tuberculosis	Tuberculin skin test result
Tuberculosis chancre	initially negative, but becomes positive during course of disease (usually after 15 days)
Tuberculosis verrucosa	strongly positive
Lupus vulgaris	usually positive
Scrofuloderma	strongly positive
Orificial tuberculosis	negative
Acute cutaneous miliary tuberculosis	negative
Papulonecrotic tuberculoid	positive
Lichen scrofulosorum	positive
Erythema induratum of Bazin	positive

**Table 3:** Histopathological features of cutaneous tuberculosis.

Different forms of cutaneous tuberculosis	Histopathological features	Observation of AFB
Well-formed granulomas with absence of caseous necrosis		
**Lupus vulgaris**	epidermis may be atrophic or hypertrophic, featuring acanthosis, papillomatosis and even pseudo-epitheliomatous hyperplasia. Presence of well-formed tuberculous granulomas accompanied more often by Langhans giant cells, or foreign body-like granulomas in the reticular dermis.	infrequent
**Lichen scrofulosorum**	non-caseating, epithelioid cell granulomas in upper dermis and around dermal appendages	not seen

Intermediate forms: granulomas with caseous necrosis		
**Tuberculosis verrucosa cutis**	marked pseudoepitheliomatous hyperplasia of the epidermis with hyperkeratosis and dense inflammatory cell infiltrate consisting of neutrophils, lymphocytes, and giant cells. The presence of granulomatous infiltrates is a cardinal sign	can be seen
**Primary cutaneous tuberculosis**	it varies according to the time of inoculation; in recent lesions there is the presence of necrotizing neutrophilic infiltrate with numerous AFB. At a later stage there is organization of granulomas	decreased number
**Acute miliary tuberculosis**	skin consists of areas of an inflammatory infiltrate composed of lymphocytes, plasma cells, and neutrophils with focal superficial dermal areas of necrosis and abscess formation without true caseating granuloma. The presence of acid-fast bacilli with vascular thrombi is characteristic of these lesions	can be seen
**Tuberculosis orificialis**	there are tuberculoid granulomas, around a median, central, and superficial ulcer accompanied by caseous necrosis in the deep dermis	not usually found
**Papulonecrotic tuberculid**	lesions showed psoriasiform epidermal hyperplasia, and epithelioid granulomas with lymphocytes and Langhans giant cells with variable amounts of necrosis seen in the upper and mid dermis with a perifollicular distribution	not usually found

Poorly formed granulomas with intense caseous necrosis		
**Scrofuloderma**	Massive central necrosis with abscess formation and in many cases, suppuration, traces of granulomas can be observed at periphery of the lesions	may be found
**Metastatic abscesses and gumma**	Central ulceration with abundant caseous necrosis, surrounded by a rim of giant cells and macrophages can be observed	frequently detected

**Table 4:** Sensitivity and specificity of PCR in the diagnosis of cutaneous tuberculosis (literature review).

References and date	No. of samples	Sensitivity (%)	Specificity (%)
(Lee et al. 2016)	574	51.1	86.3
(Tan et al. 2001)	105	100	100 (multi-bacillary form)
		Overall 73 (positivity of 55% in cases of tuberculosis verrucosa and 60% in cases of lupus vulgaris; positivity of 54% for cases of erythema induratum)	not calculated (pauci-bacillary form)
(Chawla et al. 2009)	104	74.1	96.1
(Agarwal et al. 2017)	70	24.5	not calculated
(Salian et al. 1998)	60 (formalin fixed paraffin embedded)	73.6	100
(Ogusku et al. 2003)	37	43.7	90.4
(Negi et al. 2005)	37	95.2	100
(Abdalla et al. 2009)	34	88	83
(Hsiao et al. 2003)	34	56	not calculated
(Lall et al. 2017)	31	25.8	not calculated
(Khosravi et al. 2006)	30 (formaline fixed)	75	not calculated
(Ramam et al. 2013)	28	25	73.7
(Khine et al. 2017)	25	52	not calculated
(Quiros et al. 1996)	20	85	not calculated

## Abbreviations

AFB: Acid-fast bacilli
MDR-TB: Multiple drug resistant tuberculosis
MIRU-VNTR: Mycobacterial Interspersed Repetitive Unit-Variable Number Tandem Repeat
Mtb: * Mycobacterium tuberculosis*
NTP: National Tuberculosis control Programme
PCR: Polymerase chain reaction
TST: Tuberculin skin test
XDR-TB: Extensively drug resistant tuberculosis.
